# Molecular characterization of a cellulose synthase gene (*AaxmCesA1*) isolated from an *Acacia auriculiformis* x *Acacia mangium* hybrid

**DOI:** 10.1007/s11105-012-0499-2

**Published:** 2012-08-23

**Authors:** Seok Yien Christina Yong, Ratnam Wickneswari

**Affiliations:** 1 Department of Biology, Faculty of Science, Universiti Putra Malaysia, 43400 UPM Serdang, Selangor Darul Ehsan Malaysia; 2 School of Environmental and Natural Resource Sciences, Faculty of Science and Technology, Universiti Kebangsaan Malaysia, 43600 Bangi, Selangor Darul Ehsan Malaysia

**Keywords:** *Acacia* hybrid, Cellulose synthase gene, Conserved motif, Relative gene expression, Isoform, Promoter region

## Abstract

Cellulose is the major component of plant cell walls, providing mechanical strength to the structural framework of plants. In association with lignin, hemicellulose, protein and pectin, cellulose forms the strong yet flexible bio-composite tissue of wood. Wood formation is an essential biological process and is of significant importance to the cellulosic private sector industry. Cellulose synthase genes encode the catalytic subunits of a large protein complex responsible for the biogenesis of cellulose in higher plants. The hybrid *Acacia auriculiformis* x *Acacia mangium* represents an important source of tree cellulose for forest-based product manufacturing, with enormous economic potential. In this work, we isolate the first cellulose synthase gene, designated *AaxmCesA1*, from this species. The isolated full-length *AaxmCesA1* cDNA encodes a polypeptide of 1,064 amino acids. Sequence analyses revealed that *AaxmCesA1* cDNA possesses the key motif characteristics of a CesA protein. AaxmCesA1 shares more than 75 % amino acid sequence identity with CesA proteins from other plant species. Subsequently, the full-length *AaxmCesA1* gene of 7,389 bp with partial regulatory and 13 intron regions was also isolated. Relative gene expression analysis by quantitative PCR in different tissues of the *Acacia* hybrid, suggests the involvement of the *AaxmCesA1* gene in primary cell wall synthesis of rapidly dividing young root cells. Similarity analyses using Blast algorithms also suggests a role in primary cell wall deposition in the *Acacia* hybrid. Southern analysis predicts that *AaxmCesA1* is a member of a multigene family with at least two isoforms in the genome of the *Acacia* hybrid.

## Introduction

*Acacia* is a genus of shrubs and trees consisting of approximately 1,400 species found worldwide, with more than 900 species native to Australia. *Acacia* species spread around the tropical-warm–temperate regions of both hemispheres, including Southern Asia, Africa and the Americas (Maslin et al. 2003). Many Australia tropical acacias have substantial commercial importance for the pulp and timber industries, particularly in Southeast Asia. The main *Acacia* species with high commercial values are *Acacia mangium*, *A. auriculiformis*, *A. crassicarpa* and the more recently emerging *A. auriculiformis* x *A. mangium* hybrid (McDonald et al. 2001). In Malaysia, the first natural hybridization of *Acacia auriculiformis* x *A*. *mangium* hybrid (hereafter *Acacia* hybrid) was observed in an *Acacia* plantation in Ulu Kukut, Sabah in 1971 (Tham 1976). Better stem straightness compared to *A*. *mangium* was found in the hybrid; the hybrid also inherits better self-pruning ability, stem roundness and disease resistance than *A. auriculiformis*. The hybrid also possesses intermediate characteristics between the two parental *Acacia* species (Bowen 1981) and appears to have higher cellulose and lower lignin contents, which give better pulp yields than both parental trees (Yamada et al. 1990).

Cellulose is the major component in wood, which is present universally in plant cell walls and provides mechanical strength to the plant. Cellulose accounts for about 20 % and 50 % of the primary and secondary cell walls in higher plants, respectively. Membrane-bound cellulose synthase enzyme complexes (CelS) are believed to play a key role in the biosynthesis of cellulose (Saxena and Brown 2005). These complexes are discernible as hexameric rosettes under freeze-fracture electron microscopy and consist of 36 cellulose synthase (CesA) proteins per rosette (Doblin et al. 2002; Saxena and Brown 2005; Somerville 2006). Since the first isolation of a plant *CesA* gene from a cotton tree 16 years ago, many similar attempts have been made in numerous plants with the aim of understanding the role of* CesA* genes and the mechanisms involved during cellulose biosynthesis. Previous studies revealed that the catalytic subunits of CelS in many plant species are encoded by a family of genes or isoforms (Richmond and Somerville 2000; Djerbi et al. 2005). Isoforms are alternative forms of a gene generated through splicing (Wang et al. 2012) and are found commonly in many plant species. Isoforms with differential expression patterns in different types of tissues throughout different plant developmental stages have also been reported (Hayashi et al. 2005; Joshi et al. 2004; Nairn and Haselkorn 2005; Roberts et al. 2004; Taylor et al. 2003). Mutant analyses conducted on ten *CesA* genes from *Arabidopsis thaliana* (*AtCesAs*) also revealed a number of glycosyltransferases encoded by these *AtCesAs* that function in cellulose biosynthesis in primary and secondary cell walls (Burn et al. 2002; Taylor et al. 1999; Turner and Somerville 1997). A study on *Eucalyptus* had also identified several *CesAs* associated with primary and secondary cell wall formations (Ranik and Myburg 2006). Cellulose biogenesis is a complex process, which still requires ongoing study to comprehend completely its pathways and mechanisms. Even with the completion of the *Arabidopsis thaliana* genome sequencing project, researchers still struggle to understand the biogenesis of cellulose in *A. thaliana*, let alone other plants that are less well studied.

Despite being cultivated for its timber, as well as for the pulp and paper industries, not much is known about the genetics underlying cellulose biosynthesis in the *Acacia* hybrid. Here, we report the first isolation and characterization of a cellulose synthase gene from the commercially valuable *Acacia* hybrid with the aim of understanding the deposition of cellulose in its primary and secondary cell walls.

## Materials and Methods

### Plant Material and Total RNA Preparation

Young leaves, a mixture of phloem and xylem (hereafter referred to as inner bark), flowers and greenish seedpod tissues were collected from a 7-year-old *Acacia* hybrid tree (AaHyF_1_113) planted in Plot W, Plant Biotechnology Laboratory, Universiti Kebangsaan Malaysia. Young root tissue was obtained from tissue-cultured *Acacia* hybrid (Clone M5) provided by the Forest Research Institute Malaysia. Tissues were immersed in liquid nitrogen immediately upon collection. Total RNA was extracted from the five tissues using a RNeasy Plant Mini Kit (Qiagen, Hilden, Germany) according to the manufacturer’s recommendations with minor modification, where 1 % polyethylene glycol 6000 (PEG 6000) was added to the protocol to enhance total RNA yield. Additional on-column DNase digestions were performed twice using RNase free DNase I (Promega, Madison, WI). The concentration and quality of the total RNA isolated was estimated from the ratio of the absorbance measured at 260/280 nm, and by gel electrophoresis. cDNA was synthesized using Superscript II Reverse Transcriptase (Invitrogen, Carlsbad, CA) and cDNA quality was assessed by end point reverse transcription PCR using gene specific primers (GSP).

### Relative Gene Expression Analysis by Quantitative PCR

GSP for *AaxmCesA1* and actin genes were designed based on the 3′ EST sequences identified from our *Acacia* hybrid EST dataset (Yong et al. 2011), using Primer Premier 5 (http://www.PremierBiosoft.com) and Primer 3 (http://frodo.wi.mit.edu/cgi-bin/primer3) software, respectively. The primer sequences for *CesA* and actin genes were: CesAForw1: 5′ CATACCCGTTTGTCGTCC, CesARerv1: 5′ CATCAACATTCATCCTCATTG and ActinForw: 5′ GGTAACATTGTCCTCTGTGGT, ActinRerv: 5′ CATCGTATTCTGCCTTCGA. The relative expression level of *AaxmCesA1* gene was investigated in the inner bark, young seedpod, leaf, young root and flower tissues, with the inner bark tissue as calibrator sample. PCR reactions were carried out using SYBR Green Supermix (Bio-Rad, Hercules, CA) in a iCycler iQ5 Real-Time PCR Detection System (Bio-Rad). PCR was initiated with a pre-denaturation step at 95 °C for 4 min, followed by 60 cycles of denaturation, annealing and extension at 95 °C/15 s, 55–60 °C/30s, 72 °C/15 s, respectively, then a final extension at 72 °C for 4 min. All standard dilutions, controls and samples were performed in four replicates. Standard curves for *AaxmCesA1* and *actin* were constructed using 5-fold serial dilution of the RT product synthesized from inner bark tissue. The 10μL quantitative PCR (qPCR) reactions contained a final concentration of 1x SYBR Supermix, 0.2 μm of each primer and 10 ng template cDNA. Melt curve analysis was carried out from 55 °C to 95 °C at the end of the amplification to confirm that the individual qPCR product corresponded to a single homogeneous DNA species. qPCR products were also sequenced directly using ABI 3700 (Applied Biosystems, Foster City, CA) at Macrogen Corporation, Korea, to ensure amplification specificity.

### Isolation of Full-Length *AaxmCesA1* cDNA

Total RNA was extracted from the leaf samples of a single *Acacia* hybrid individual plant (HyAa/F1 113) using an RNeasy Plant Mini Kit (Qiagen). The full-length *AaxmCesA1* cDNA was isolated using a BD SMART™ RACE cDNA amplification kit (Clontech, Palo Alto, CA) according to manufacturer’s instructions. RACE primers were designed using Primer Premier 5 software. The primers of RamA1: 5′ CCTTCCGAGCAGACCCTTCA, RCesA1-1Rb: 5′ GCCTGCTAAGGAAAATCTCAATGGACC and NRA11R: 5′ GCAATAGGCACCACAGACTCAAAGG were used for 5′ RACE PCR; FAMA1: 5′ GGTGGCATTCCTCCCTCAAC was used for 3′ RACE PCR. Each RACE-PCR reaction containing 2.5μL first strand cDNA, 1x UPM A Mix, 0.2 μM GSP and 41.5μL master mix was performed using the Mastercycler PCR machine (Eppendorf, Hauppauge, NY). PCR was performed using a touch down cycling profile starting with 5 cycles of amplification at 94 °C/30s, 72 °C/3 min, followed by another 5 cycles at 94 °C/30s, 70 °C/30s, 72 °C/3 min, then 25 cycles at 94 °C/30s, 68 °C/30s, 72 °C/4 min, and finally an extension at 72 °C for 16 min. RACE PCR products were electrophoresed on 1.2 % agarose gel in 1 x TAE buffer at 80 V, gel purified using Qiaquick Gel extraction kit (Qiagen) and cloned in the PGEM-T easy Vector system (Promega), before sequencing from both ends using ABI 3700 (Applied Biosystems) at Macrogen Corporation, Korea.

### Isolation of *AaxmCesA1* Intron Regions

Genomic DNA was extracted from the young leaf of an individual *Acacia* hybrid (AaHy113F_1_) using the CTAB method (Doyle and Doyle 1990). DNA sequences obtained from our RACE PCR were used to design primers (Table 1) using Primer Premier 5 and Primer 3 software for the amplification of the intron regions. Each 50μL PCR reaction contained 20–40 ng genomic DNA, 1x PCR buffer, 10 mM dNTP mix, 5uM of each primer and 1 U Hot Start* Taq* polymerase. PCR was performed in Mastercycler PCR machine (Eppendorf) using a hot start cycling profile, which starts with an initial denaturation at 96 °C for 4 min, followed by 25 cycles of amplification at 96 °C/30 s, 64–69.9 °C/30 s, 72 °C/1 kb min^−1^, and a final extension at 72 °C for 16 min. PCR products were gel purified and sequenced from both ends at Macrogen Corporation, Korea.

**Table 1:** Gene specific primers (GSP) used in intron isolation

Primer pair	GSP	Primer sequence (5′—3′)	Annealing temperature (°C)	Product size (bp)
1	FamCes	GGTGGCATTCCTCCCTCAAC	64	897
	RamCes	CCTTCCGAGCAGACCCTTCA	64	
2	*CesA*1-F2	GAAGAGGGCAGCAAAAAGAAC	60	648
	*CesA*1-R2	GGGAGCACACAGTAAGCAATC	60	
3	A1WF8	CCAATCTGTGCGAACTAC	48.3	1,050
	A1WF8b	CCTCCACTATGACCTAAG	43.7	
4	A1WF9	GGAAGGGGATGACGATG	52.8	1,013
	AIWR9	GCAATAGGCACCACACG	52.7	
5	A1WF10	GAGCCAAGAGCCCCTGA	55.9	1,782
	A1WR10	GCCCACCGAAGCACCT	56.5	
6	A1WF11	GAAAAGGTTCACTCAC	36.7	504
	A1WR11	TAACAACACGATAAGG	36.6	
7	A1WF17	TTGTTTATGTTTCTCGTG	49.2	1,534
	A1WR17	TGAGGTAAAGGGGTAGA	49.2	
8	*CESA*1In1F	CTGGGGAATGGTGGCTGG	65	1,000
	*CESA*1In1R	TTCATAACAAGGACGACAAACTGGGTA	65	
9	*CESA*1In2F	CATACCCAGTTTGTCGTCC	56	1,420
	AIWR9	TCATCCTCATCGTCATCCC	56	

### Isolation of *AaxmCesA1* Promoter Region

The promoter sequence was isolated using the genome walking approach described by Siebert et al. (1995). Two primers, CesAPr2: 5′ ATACGACCCAGCCACCATTCCCACAGT and CesANPr2: 5′ GAAGAAGACAGTGAGAGAGATTTGGAGC were designed using Primer Premier 5 software. Genomic DNA was extracted from the young leaf of an individual *Acacia* hybrid (AaHy113F_1_) using the CTAB method (Doyle and Doyle 1990). Isolated genomic DNA was digested with six restriction enzymes: *Bsr*BI, *Nru*I, *Hpa*I, *Bst*Z17I, *Fsp*I and *Sna*BI (New England Biolabs, Hitchin, UK) to produce blunt-end fragments that were then ligated to genome walker adapter. Primary PCR was performed with primer CesAPr2, and then the 10x diluted primary PCR product was used as template for the secondary ‘nested’ PCR with primer CesANPr2. Each PCR reaction contained a final concentration of 1× PCR buffer, 1.5 mM MgCl_2_, 0.4 μM of each adaptor and gene specific primer and 2 U Hot start *Taq*, and amplification used a hot-start touch-down PCR cycling profile. Amplification started with a pre-denaturation at 96 °C for 3 min, followed by 7 cycles of amplification at (96 °C/15 s, 72 °C/3 min), then another 20 cycles at (96 °C/15 s, 68 °C/3 min) and ended with a final extension at 68 °C for 15 min. Gel-purified secondary PCR product was sequenced from both ends using ABI PRISM 3730XL DNA analyzer (Applied Biosystems) at Research BioLabs Technologies, Singapore.

### Sequence and Structure Analyses

The full-length *AaxmCesA1* cDNA sequence isolated was subjected to similarity search using Blast (http://www.ncbi.nlm.nih.gov). A total of 24 CesA protein sequences of the closest homologues were retrieved from the NCBI database (http://www.ncbi.nlm.nih.gov) for phylogenetics analysis. The accession numbers for these sequences are: AAT66940.1, XP 003522623.1, AEK31219.1, XP 002515536.1, XP 002324291.1, AAY60847.1, AAY78952.3, ACT16001.1, AAY43218.1, NP 194967.1, AAO25536.1, NP 001054788.1, NP 001105574.1, AAP97497.1, AAF89963.1, XP 002880684.1, NP 180124.1, NP 001051648.1, NP 001105621.1, ADZ16121.1, ADZ16119.1, XP003540527.1, AAT66941.1, and AAY43219.2. In addition to these 24 sequences, selected CesA protein sequences from *Arabidopsis* (NP 195645.1, NP201279.1, NP 196136.1, Q84JA6, Q8L778, NP 197244.1 and NP 567564 .1), *Hordeum vulgare* (AY483150, AY483152, AY4831555), *Oryza sativa* (BAD30574), *Zea mays* (NP_001104954.1, NP_001104959.1), *Gossypium hirsutum* (U58283), *Bambusa oldhamii* (AAY43221.1) and *Eucalyptus grandis* (AAY60843.1, AAY60845.1, AAY60846.1 and AAY60848.1) were included. Multiple sequences alignment was performed using ClustalW2 (http://www.ebi.ac.uk/Tools/es/cgi-bin/clustalw2) and phylogenetic analysis was carried out using MEGA Version 5 (Tamura et al. 2011). The unrooted phylogenetic tree was constructed using the neighbor-joining method based on distance matrices with 1,000 bootstrap replicates and the Jones-Taylor-Thornton model.

Conserved domains were detected using Conserved Domain software (http://www.ncbi.nlm.nih.gov/Structure/cdd/wrpsb.cgi), ClustalW2 and Interproscan software (http://www.ebi.ac.uk/Tools/cgi /iprscan.cgi). Promoter sequence was analyzed using Prediction of Plant Promoter (http://www.softberry.com/berry.phtml) and Neural Network Prediction (http://www.fruitly.org/seq_tools/promoter.html) software. The cDNA, introns and promoter sequences amplified from RACE-PCR, primer walking and genome walking respectively, were analyzed using CAP contig assembly (Huang and Madan 1999) and ClustalW2 to predict the *AaxmCesA1* full-length gene sequence. The software “Finding 5′, internal and 3′ coding exons” (http://linux.softberry.com/cgi-bin/progrmas/gfind/fex.pl) was applied to predict exon–intron boundaries. The full-length gene sequence was also compared with the full-length gene sequences of other species using ClustalW2 to predict intron and exon positions.

### Gene Isoform Analysis

Genomic DNA was extracted from young leaves of an *Acacia* hybrid individual plant (AaHy113F_1_) using the CTAB method (Doyle and Doyle 1990). Genomic DNA (15 μg) was digested with two restriction enzymes (*Bsr*BI and *Bst*Z17I; New England Biolabs) at 37 °C for 18 h. The phenol-chloroform purified digested DNA was electrophoresed on a 0.8 % agarose gel in 1x TAE buffer at 25 V for 16 h and transferred to Hybond N + filter membrane (Amersham, Little Chalfont, UK) by downward capillary transfer (Chomczynski 1992) in alkaline buffer (Sambrook and Russell 2001). The blot was hybridized with a 1.6 kb-*AaxmCesA* probe labeled with ^32^P radioisotope deoxycytidine 5′-Triphosphate (dCTP) disodium salt using Megaprime DNA Labelling Systems (Amersham), and unincorporated probe was filtered on a Sephadex G-75 column. Hybridization and washing were carried out according to standard procedures described in Sambrook and Russell (2001).

## Results

### Relative Gene Expression Analysis

**Fig. 1:**
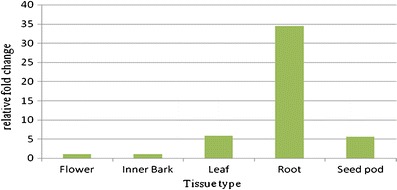
Relative expression level of *AaxmCesA1* in five different tissues of the *Acacia* hybrid. The relative fold change of gene expression in flower, inner bark, leaf, root and seed pod tissues are presented with inner bark as the reference sample

The expression levels of Aaxm*CesA1* in young root, inner bark, young leaf, greenish seedpod and flower tissues were investigated using qPCR. Melt curve analysis showed a single peak for the amplification of *AaxmCesA1* gene denoting PCR amplification specificity. Direct sequencing of the qPCR products also confirmed the originality of the amplified products. The highest level of transcript abundance was observed in young root tissue whereas the lowest was evident in inner bark tissue. The expression levels of the *AaxmCesA1* gene in the *Acacia* hybrid were found in a decreasing pattern from leaf, seed pod to flower (Fig. 1). However, statistical analysis using independent t-test at 95 % confidence interval shows that the differences in the expression levels between inner bark tissue and all other tissues studied were not statistically significant with the exception of young root tissue.

### Full-length *AaxmCesA1* cDNA

**Fig. 2:**
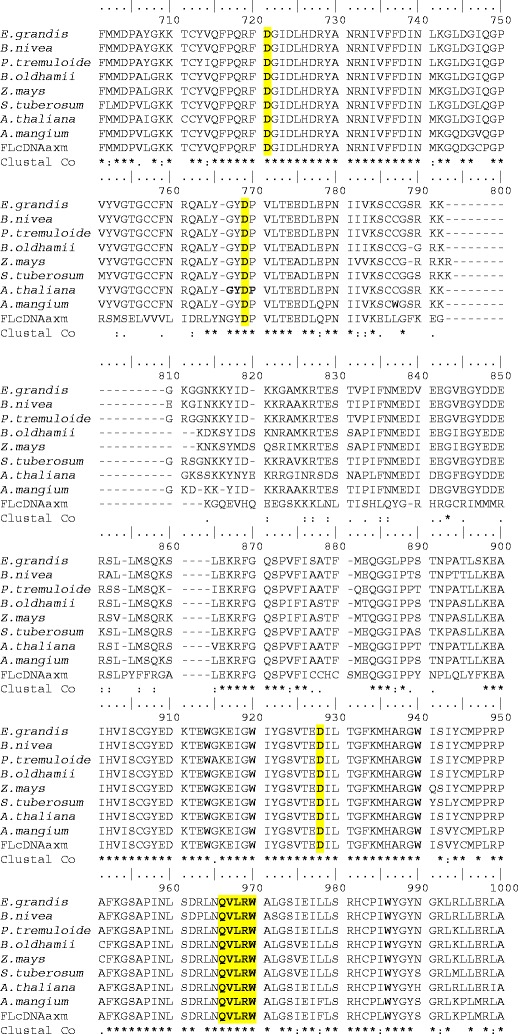
Comparison of the partial amino acid sequence of *Acacia* hybrid (labelled FLcDNAaxm) with other plant CesAs using ClustalW. The conserved amino acids/motifs D, D, D, and QXXRW (highlighted in *yellow*) are found in all the *CesAs* of the different species. Identical amino acid residues are indicated with* asterisks*. Accession numbers for the sequences are as follows: *E*. *grandis* (gb|AAY60847.1), *B*. *nivea* (gb|AAY43218.1), *B*. *oldhamii* (gb|aay43218.1), *P*. *tremuloides* (gb|AAO25536.1),* Z. mays* (ref|NP_001105574.1), *S*. *tuberosum* (gb|AAP97497.1), *A*. *thaliana* (NP_19496.1), *A*. *mangium* (gb|AAT66940.1)

The isolated full-length cDNA of the *AaxmCesA1* gene is 3,835 bp long, which includes the 3′ UTR (437 bp) and 5′UTR (212 bp) regions. The predicted open reading frame is 1,062 residues in length and encodes a protein of 146 kDa with a predicted p*I* of 8.84. Multiple alignment analysis performed on the deduced amino acid sequences of the *Acacia* hybrid and CesA protein sequences of other species revealed a high degree of amino acid similarity throughout most of the sequences length, diverging mostly at the N and C-terminal ends and hypervariable regions (HVR). Although the various CesA proteins from different species varied in their degree of sequence similarity, a conserved motif (D, D, D, QXXRW) was found in all sequences analyzed (Fig. 2). The first, second, third D and QXXRW were found at positions 710, 758, 891 and 929 residues of the translated protein sequence of the *Acacia* hybrid, respectively.

**Fig. 3:**
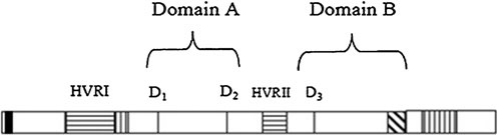
Simplified schematic representation of the major regions of the AaxmCesA1 protein.* Horizontal shading* Hypervariable regions (*HVRI, HRVII*),* vertical shading* putative transmembrane regions,* D*_*1*_–*D*_*3*_ conserved aspartic residues,* diagonal shading* conserved QXXRW motif,* black vertical bar* zinc finger domain

Two HVRs were also predicted from the deduced amino acid sequence of AaxmCesA1. HVRI was found before the first aspartic acid in Domain A and HVRII between the second aspartic acid in Domain A and third aspartic acid in Domain B (Fig. 3). HVRI was predicted at positions 201–330, and HVRII at positions 760–830. Other domains, including pfam cellulose_synt, which was identified in the region between residues 394 and 1126, glycosyltransferase family_2 at residues 882–1020, COG1215 (glycosyltransferase) at residues 882–1048 and bcsA at residues 880–1011, were found in the deduced amino acid sequence of the *Acacia* hybrid. Interpro Scan performed on the full-length *AaxmCesA1* cDNA identified additional domains, namely zinc fingers (RING/FYVE/PHD-type) and transmembrane regions. Two putative zinc finger domains were predicted at residues 82–160 and 161–200 in the *Acacia* hybrid amino acid sequence. Two transmembrane domains were found in the amino terminal region and another six in the carboxy terminal region. The first two transmembrane domains were located before the beginning of the globular domain A at positions 361–381 and 390–410. The other six transmembrane domains were located just after globular domain B at positions 933–953, 963–983, 1002–1020, 1035–1053, 1116–1138 and 1249–1269.

Blast X analysis revealed significant matches between the deduced AaxmCesA1 protein sequence and the CesA protein sequences of *A. mangium* (AAT66940.1), *Eucalyptus* spp (AEK31219.1; AAY60847.1), *Bohmeria nivea* (AAY78952.1), *Populus* spp (XP_002308657.1; AAO25536.1), *Arabidopsis thaliana* (NP_194967.1), *Bambusa oldhamii* (AAY43218.1; AAY43216.1) and *Ricinus communis* (XP_002515536.1), with *E*-value = 0 and amino acid identity of more than 70 %. Based on the predictions of Blast N analysis, the *AaxmCesA1* cDNA shares 95 % nucleotide sequence similarity with *Acacia mangium CesA1* (gb|AY643519.1) at *E* value = 0. It also exhibits over 75 % nucleotide sequence similarity with *Ricinus communis CesA6* (ref|XM002515490.1), *Glycine max CesA1* (XP_003522623.1), *Populus tremuloides CesA4* (gb|162181.1), *Populus trichocarpa CesA* (ref|XM_002308621.1), *Eucalyptus camaldulensis CesA5* (gb|HQ864587.1) and *Arabidopsis thaliana CesA1* (ref|NM_119393.2). The high level of similarities in the nucleotide and protein sequences of these *CesA* genes suggest an orthologous relationship among them. Blast analyses also suggested a possible role for the *AaxmCes1* gene in primary cell wall formation of the *Acacia* hybrid as most of the matched CesA proteins with known function from other species are related to primary cell wall formation.

**Fig. 4:**
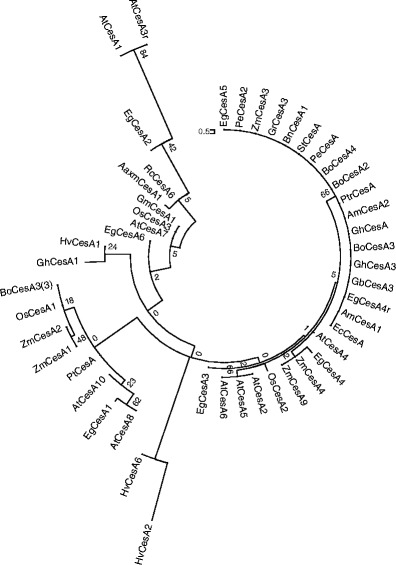
Unrooted NJ tree with 1,000 bootstrap replicates derived from the alignment of deduced amino acid sequences of AaxmCesA1 with 43 CesA protein sequences (AAT66940.1, XP 003522623.1, AEK31219.1, XP 002515536.1, XP 002324291.1, AAY60847.1, AAY78952.3, ACT16001.1, AAY43218.1, NP 194967.1, AAO25536.1, NP 001054788.1, NP 001105574.1, AAP97497.1, AAF89963.1, XP 002880684.1, NP 180124.1, NP 001051648.1, NP 001105621.1, ADZ16121.1, ADZ16119.1, XP003540527.1, AAT66941.1, AAY43219.2, NP 195645.1, NP201279.1, NP 196136.1, Q84JA6, Q8L778, NP 197244.1, NP 567564 .1, AY483150, AY483152, AY4831555, BAD30574, NP_001104954.1, NP_001104959.1, U58283, AAY43221.1 AAY60843.1, AAY60845.1, AAY60846.1 and AAY60848.1). Aaxm, *Acacia* hybrid; Am, *Acacia mangium*; At, *Arabidopsis thaliana;* Eg, *Eucalyptus grandis*; Os, *Oryza sativa*; Zm, *Zea mays*; Ptr, *Populus tremulodes*; Gh, *Gossypium hirsutum*; Bo, *Bambusa oldhamii*; Pe, *Phyllostachys eduli*; Rc, *Ricinus communis*; Hv, Hordeum vulgare L.; Gm, *Glycine max*; Ec, *Eucalyptus camaldulensis*; Bn, *Bohmeria nivea*; St, *Solanum tuberocum*; Gb, *Gossypium barbadense*; Gr, *Gossypium raimondii*; Pt, *Populus trichocarpa*

A total of 43 CesA amino acid sequences from other species, in plants ranging from dicots, monocots, angiosperms to gymnosperms, were retrieved from the NCBI website (http://www.ncbi.nlm.nih.gov) and compared with the AaxmCesA1 predicted protein sequence. Figure 4 shows an unrooted tree constructed based on neighbor-joining analysis for the deduced amino acid of AaxmCesA1 with the 43 protein sequences of other plant species. Although not all the *CesA* genes of these plants have yet been identified, a preliminary conclusion that can be drawn is that, as in most large gene families, orthologous genes are more similar than paralogous genes. Thus, many of the groups in this analysis contain members from plants of both monocot and dicot or angiosperm and gymnosperm lineages.

### Full-Length *AaxmCesA1* Gene

A total of nine primer pairs were used to isolate all the intronic regions of the *AaxmCesA1* gene. The alignment and assembly of the full-length cDNA sequences obtained from RACE-PCR, and the sequences of introns amplified through primer walking, resulted in a partial gene sequence of 6,545 bp with 13 predicted introns. The gene structure was predicted based on multiple alignment analysis of the full-length *AaxmCesA1* gene sequence with the *CesA* full-length gene sequences of *A. thaliana CesA1* (NM119393.2) and *CesA10* (128111.2) and full-length cDNA sequences of *A. mangium* (AY643519) and *E. grandis* (DQ014509.1). Prediction of these 13 introns was also supported by “Finding 5′, internal and 3′ coding exons” software.

A 1-kb fragment was amplified successfully from *Acacia* hybrid genomic DNA digested with *Hpa*I using the genome walking approach, which yielded a partial promoter region of 844 bp. Promoter Prediction and Neural Network Prediction software predicted several core promoter elements from the partial nucleotide sequence of the promoter region isolated in this study. The transcription start site (TSS) was found at position 840 bp. Three core elements of the promoter, namely TATA, CAAT and GC boxes were predicted at −32, −53 and −151 bp, respectively, upstream of the TSS of full-length *AaxmCesA1* gene. A full-length *AaxmCesA1* gene of 7,389 bp was then obtained by assembling the promoter sequence with the partial *AaxmCesA1* gene sequence. The isolated *AaxmCesA1* consists of 40 % GC and 60 % AT. Blast N analysis of the full-length *AaxmCesA1* gene exhibited a significant match to the full-length *AtCesA1*gene (ref|NC 003075.7) located on chromosome 4 and the *AtCesA10* gene located on chromosome 2 of the *A. thaliana* genome, with *E*-values of 3e^−149^ and 0, respectively.

**Fig. 5:**
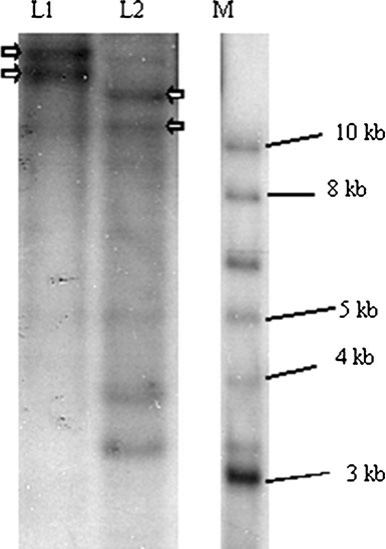
Southern blot analysis. Genomic DNA isolated from leaves of the *Acacia* hybrid was digested with *BstZ*17I or *Hpa*l, and hybridized with a ^32^P-labeled 1.6 kb partial fragment from the *AaxmCesA* gene. Lanes:* L1* *BstZ*17I-digested DNA,* L2* *Hpa*l-digested DNA,* M* 2 log DNA ladder

Southern blot analysis using genomic DNA digested with restriction enzymes *BstZ*17I and *Hpa*l revealed several hybridization bands (Fig. 5). Knowing that most *CesA* genes in plant species are larger than 4 kb, the hybridization pattern in the Southern analysis suggested that the *CesA* gene family might consist of up to four isoforms in the *Acacia* hybrid genome. However, two of these hybridization bands (indicated by arrows in Fig. 5) showed higher intensity than other bands in both digested genomic DNA samples. Based on this, we are confident that there are at least two *AaxmCesA* isoforms present in the genome of the *Acacia* hybrid. As the 1.6 kb *AaxmCesA*1 probe included the HVRII region in its design, sequence variation might cause failure of the probe to hybridize and detect all the different isoforms. This also reflects the possible high sequence variation in the HVRII region of the *AaxmCesA* genes of the *Acacia* hybrid but further investigation is necessary.

## Discussion

Isolation of a candidate *CesA* gene is a fundamental step towards understanding the gene functions and mechanisms involved during cellulose biogenesis. Although mutant analyses conducted on *Arabidopsis* have provided significant information on gene function, we are still far from understanding the complete pathway of cellulose biosynthesis in plants. Furthermore, detailed information on the *CesA* gene family is available only from a limited number of plants, such as barley (Burton et al. 2004), *Arabidopsis* (Richmond 2000) and poplar (Djerbi et al. 2004). In the current study, we have isolated and characterized the *AaxmCesA1* gene from the *Acacia auriculiformis* x *mangium* hybrid for the first time.

Cellulose is the main component in cell wall formation and is found in all plant tissues, but the deposition of cellulose in different cell types varies (Delmer and Amor 1995). In our analysis, qPCR relative gene expression patterns revealed that *AaxmCesA1* is expressed in all the tissues investigated but at different levels. The higher levels of transcript abundance observed in young root tissue most probably demonstrates an active involvement of this *AaxmCesA1* in the primary cell wall formation of actively dividing young root tissue. Fagard et al. (2000) and Wang et al. (2001) also showed that some *CesAs* acted early in the process of root hair outgrowth and elongation. The varying expression patterns of this *AaxmCesA1* in the five tissues tested here suggested the possibility that more than one *CesA* is needed for the biosynthesis of cellulose in primary and secondary cell walls in the *Acacia* hybrid. Previous studies have proposed the involvement of at least three types of *CesA* isoforms namely, α_1_, α_2_ and β, in the spontaneous arrangement of *CesA*s in the cellulose synthase complex of higher plants (Ding and Himmel 2006; Doblin et al. 2002). In *Arabidopsis*, a cellulose synthase complex consisting of three unique subunits (*AtCesA1*, *AtCesA3* and *AtCesA6*) was required for primary cell wall synthesis (Persson et al. 2007). Samuga and Joshi (2002) also showed that two subunits viz., *PtrCesA1* and *PtrCesA2* are involved in secondary cell wall formation in *Populus tremuloides*. Another study conducted on the *CesA* genes of *Oryza sativa* revealed that *OsCesA*1, *OsCesA3*, *OsCesA8* and *OsCesA4*,* OsCesA7*,* OsCesA9* are strongly co-expressed in tissue of typical primary and secondary cell walls respectively. These findings suggest that each set of these *OsCesA* subunits belongs to the same cellulose synthase complex, which catalyze similar cellulose biosynthesis (Wang et al. 2010; Tanaka et al. 2003). Different types of *CesA* isoforms have also been found to be co-expressed in barley; *HvCesA1*, *HvCesA2* and *HvCesA6* (Burton et al. 2004) and *Eucalyptus*; *EgCesA1, EgCesA2* and *EgCesA3* (Ranik and Myburg 2006). Thus, it is very likely that we will find other co-expressing *CesA* isoforms responsible for the biogenesis of cellulose in either primary or secondary cell wall in the *Acacia* hybrid.

Based on our Blast analysis, we propose a role in primary cell wall synthesis for the isolated *AaxmCesA1*, concurring with our relative gene expression study. Blast analyses revealed a high degree of amino acid sequence similarity between the AaxmCesA1 isolated in this study and GmCesA1, EgCesA5 (Ranik and Myburg 2006), OsCesA1 (Wang et al. 2010), BoCesA2 (Chen et al. 2010), AtCesA1 (Desprez et al. 2007) and AmCesA1. The high degree of amino acid sequence similarity suggests that *AaxmCesA1* might be orthologous to *EgCesA5*,* AtCesA1*,* BoCesA2* and* OsCesA1*, which are all known to be associated with primary cell wall formation. Mutant analysis performed on *Arabidopsis* in past study showed that a single amino acid substitution in *AtCesA1* resulted in a marked reduction of crystalline cellulose synthesis, and the disassembly of cellulose synthase complexes (Arioli et al. 1998). This finding suggests that *AtCesA1* is involved in primary cell wall synthesis in *Arabidopsis*.

The length (3,835 bp) of the *AaxmCesA1* cDNA isolated in the current study is similar to that of *A. thaliana* (3*–*3.5 kb; Richmond 2000) and poplar (3–3.3 kb; Joshi et al. 2004). The D, D, D, QXXRW signature motif predicted from the deduced amino acid sequence of the *A*cacia hybrid is a strong predictor for family 2 of β-glycosyltransferases (Campbell et al. 1997). β-Glycosyltransferases can be categorized into processive or non-processive glycosyltransferases depending on the transfer of multiple or single sugar residue to an acceptor molecule. Processive glycosyltransferase is composed of two conserved domains (domains A and B), while non-processive glycosyltransferase contains only domain A (Saxena and Brown 1997). Based on this classification, the *AaxmCesA1* isolated in our study is postulated to belong to the processive class because it contains both domains A (consisting two conserved aspartic acid, D) and B (consisting one aspartic acid, D and the QXXRW sequence). The two aspartic acid residues in domain A, and a single aspartic acid residue and the sequence QXXRW in domain B are found to be strictly conserved in all the *CesA* genes analyzed here. This finding concurs with the analysis performed by Saxena et al. (1994). The distance between the first and second conserved aspartic acid in domain A of the AaxmCesA1 amino acid sequence is 48 residues, while the distance between the second aspartic acid in the domain A with the third aspartic acid in the domain B is 133 residues. A distance of 38 residues is predicted between the third aspartic residues and the QXXRW sequence (Table 2). Saxena et al. (1994) also predicted the distance between the third aspartic acid residue and the QXXRW sequence in domain B to be the most conserved. In contrast, the distance between the two conserved acid aspartic residues in domain A is the most variable, extending from 46 to 120 residues. Saxena and colleagues suggested that these aspartic acid residues might be involved in the catalytic reaction while the QXXRW region was suspected to be responsible for the processivity mechanism.

**Table 2:** Amino acid distances between conserved residues, and their position, in the deduced amino acid sequence of the *Acacia* hybrid

Conserved residues	Amino acid distance between conserved residues in *CesA* of *Acacia* hybrid (residues)	Range of amino acid distance (residues)
D_1_−D_2_	48	46–120
D_2_−D_3_	133	92–138
D_3_−QXXRW	38	33–38

The prediction of several conserved domains, including the pfam cellulose_synt, glycosyltransferase family_2, COG1215 (glycosyltransferase), bcsA, Interpro zinc finger (RING/FYVE/PHD-type) and transmembrane regions, from the deduced amino acid sequence of the *AaxmCesA1* cDNA strongly supports the view that the isolated gene is a cellulose synthase. Cellulose_synt and the glycosyltransferase domains are common to all cellulose synthase genes. Domain bcsA predicted in the AaxmCesA1 protein sequence is also one of the catalytic subunits of cellulose synthase (Wong et al. 1990). Two zinc fingers were also predicted in the N terminus of the AaxmCesA1 deduced amino acid sequence. The prediction of a zinc finger is consistent with the predicted CesA protein features of *Arabidopsis* (Richmond 2000) and poplar (Joshi et al. 2004). Kurek et al. (2002) identified two putative zinc fingers in the first conserved region of the N terminus of CesA protein in the cotton tree. The location of two zinc fingers within the cytoplasmic N-terminal region of the protein suggested their involvement in protein–protein interactions between cellulose synthase subunits (Kawagoe and Delmer 1997; Kurek et al. 2002; Richmond, 2000). These conserved zinc-finger sequences are suspected to act as redox-regulated multimerization domains that are involved in the assembly of cellulose synthase monomers into rosette complexes.

We also predicted eight transmembrane regions in the *AaxmCesA*1 amino acid sequence; the distribution of these regions is similar to that of Richmond’s prediction in *A. thaliana*. According to Richmond (2000), all members of the cellulose synthase superfamily appear to be integral membrane proteins, with one or two transmembrane domains in the amino terminal region, and three to six transmembrane domains in the carboxy terminal region of the protein. This nature of all cellulose synthase superfamily members is crucial, as cellulose is a water-insoluble polymer. Thus, cellulose cannot be synthesized inside the cells but can only be deposited on the external surface of each plant cell. These transmembrane helices are believed to be involved in creating a channel whereby the glucan chain is secreted outside the cell to form new cell walls (Kurek et al. 2002). We also found that the intron–exon organization is highly conserved among most of the *CesA* genes analyzed in this study. We predicted 13 intron regions in the full-length *AaxmCesA1* gene, which is comparable to the arrangement found in *A*. *thaliana* (Richmond 2000) and poplar (Joshi et al. 2004). The highly conserved organization of the *CesA* gene structure across species might reflect its importance for gene functionality in plants.

Analysis of the *AaxmCesA1* partial promoter sequence of 840 bp predicted several core elements. These core elements consist of TATA, CAAT and GC boxes, which are generally found in the regulatory regions of promoters and are involved in the regulation of gene expression. Little is known about the transcriptional regulation of this gene family generally, and the *Acacia* hybrid in particular. A study conducted by Creux et al. (2008) demonstrated that *CesA* promoters from the *Eucalyptus* tree are functional in *Arabidopsis* despite the low sequence similarity between them. Others reported that co-expressed genes harbor conserved transcription factor binding sites (Harmer et al. 2000). Study of the promoter region of *AaxmCesA1* is important as it provides an avenue to regulate the expression of *AaxmCesA1* in the *Acacia* hybrid. We aim to isolate the complete promoter region of the *AaxmCesA1* and investigate the regulatory mechanism of this gene in our future efforts. Isolation and mutant analyses of the promoter could provide experimental proof for the hypothetical mechanisms orchestrating the transcriptional activity of this gene.

Past investigations conducted on many plant species, including poplar, maize and cotton, have demonstrated that *CesA* belongs to a multigene family. Desprez et al. (2007) have shown that at least three *CesA* isoforms are needed for the biosynthesis of cell walls. Different isoforms have been found to be co-expressed in many plant species including rice (Tanaka et al. 2003), *Arabidopsis* (Taylor et al. 2003), poplar (Joshi et al. 2004) and barley (Burton et al. 2004). Mutant analyses of *CesA1*, *CesA3* and *CesA6* showed defects in the primary cell walls of *Arabidopsis* (Arioli et al. 1998; Fagard et al. 2000). Based on our isoform study, we conjectured that there are other *AaxmCesA* isoforms apart from *AaxmCesA1* that are responsible for the biosynthesis of cellulose in the *Acacia* hybrid. We predict that at least two isoforms of *AaxmCesAs* are present in the genome of the *Acacia* hybrid. The probe used in the isoform analysis was a PCR product amplified from the genomic DNA of the *Acacia* hybrid flanking region, and includes the conserved aspartic residues D_1_, D_2_, D_3_ and the HVRII. The conserved aspartic residues D_1_, D_2_, D_3_ enable the identification of *CesA* genes whereas the HVRII helps in differentiating the different isoforms. HVRII was designated as a class specific region (CSR) and has been used to isolate cDNAs encoding *CesAs*. This is because this region could discriminate each member of the *CesA* family efficiently (Liang and Joshi 2004; Ranik and Myburg 2006). Therefore, we expect to identify other *AaxmCesAs* and at the same time distinguish the different isoforms of the *AaxmCesA* gene family in the *Acacia* hybrid using this probe. In this current study, we failed to identify a third isoform. Nevertheless, this failure might possibly reflect high sequence variation in the HVRII region of the *AaxmCesA* genes of the *Acacia* hybrid. In a previous study conducted on aspen, Samuga and Joshi (2002) concluded that orthologous genes show a higher percentage of identity than paralogous genes. In their work, they found that CesA from the same species exhibits more amino acid sequence variation than between CesA of different species. This variation might explain why we did not detect a third isoform using this probe.

Assessment of gene function through mutant analysis and gene expression studies are important. Although remarkable progress has been made in recent decades, especially in the model plant *Arabidopsis*, towards unraveling the process of cellulose biosynthesis, many unanswered questions still remain. While mutant analysis can reveal whether a gene is critical for a particular process, it cannot provide us with information on the exact pathways or mechanisms that take place during cellulose deposition. We still have no complete picture of how plants synthesize cellulose during primary and secondary cell wall formation. In the case of the *Acacia* hybrid, only the *AaxmCesA* gene has been isolated thus far. Thus, efforts to isolate and investigate all the *AaxmCesA* genes must continue in order to obtain more a comprehensive insight into the biogenesis of cellulose in the *Acacia* hybrid. Our current report represents the first step towards achieving this goal.
